# Wheat Spike Blast Image Classification Using Deep Convolutional Neural Networks

**DOI:** 10.3389/fpls.2021.673505

**Published:** 2021-06-17

**Authors:** Mariela Fernández-Campos, Yu-Ting Huang, Mohammad R. Jahanshahi, Tao Wang, Jian Jin, Darcy E. P. Telenko, Carlos Góngora-Canul, C. D. Cruz

**Affiliations:** ^1^Department of Botany and Plant Pathology, Purdue University, West Lafayette, IN, United States; ^2^Lyles School of Civil Engineering, Purdue University, West Lafayette, IN, United States; ^3^School of Electrical and Computer Engineering, Purdue University, West Lafayette, IN, United States; ^4^Department of Agricultural and Biological Engineering, Purdue University, West Lafayette, IN, United States; ^5^Tecnológico Nacional de México/IT Conkal, Conkal, Yucatán, Mexico

**Keywords:** wheat blast, convolutional neural networks, inter-rater agreement, severity classification, plant disease phenotyping, breeding, deep learning, controlled conditions

## Abstract

Wheat blast is a threat to global wheat production, and limited blast-resistant cultivars are available. The current estimations of wheat spike blast severity rely on human assessments, but this technique could have limitations. Reliable visual disease estimations paired with Red Green Blue (RGB) images of wheat spike blast can be used to train deep convolutional neural networks (CNN) for disease severity (DS) classification. Inter-rater agreement analysis was used to measure the reliability of who collected and classified data obtained under controlled conditions. We then trained CNN models to classify wheat spike blast severity. Inter-rater agreement analysis showed high accuracy and low bias before model training. Results showed that the CNN models trained provide a promising approach to classify images in the three wheat blast severity categories. However, the models trained on non-matured and matured spikes images showing the highest precision, recall, and F1 score when classifying the images. The high classification accuracy could serve as a basis to facilitate wheat spike blast phenotyping in the future.

## Introduction

Wheat blast is an emergent disease caused by the Ascomycetous fungus *Magnaporthe oryzae Triticum* (MoT). MoT was first detected in Brazil in 1985, with successive spread to Bolivia, Paraguay, and Argentina (Igarashi et al., 1986; Barea and Toledo, 1996; Viedma, 2005; Cabrera and Gutiérrez, 2007; Perello et al., 2015). In 2016, a wheat blast outbreak was first reported in Bangladesh, apparently due to the unintended importation of MoT-infected South American grain (Aman, 2016; Malaker et al., 2016). Many countries in South Asia are actively monitoring wheat fields for the presence of MoT (Bhattacharya and Pal, 2017; Mottaleb et al., 2018). In 2020, MoT presence was reported in Zambia, Africa, which summates another continent to the list (Tembo et al., 2020). Cruz et al. (2016b) predicted areas at risk in the United States (southern and pacific northwest states) for MoT establishment and the threat of this pathogen to soft- and hard-red winter wheat production.

MoT can infect leaves, stems, and seeds, although the most remarkable and studied symptoms are associated with the spike (Igarashi et al., 1986; Cruz et al., 2015; Cruz and Valent, 2017; Ceresini et al., 2019). The infection by MoT of the spike, spikelets, or rachis causes the wheat spike blast, inducing partial or complete bleaching of the spikes (Igarashi, 1986). Infection can cause shriveled grain reducing the grain quality and yield. A wide range of disease intensities can occur depending on the susceptibility of cultivars planted and the prevalent weather conditions (Goulart and Paiva, 1992).

Warm temperatures, excessive rain, long and frequent spike wetness, and limited fungicide efficacy exacerbate the intensity of wheat blast epidemics, especially in susceptible cultivars (Goulart et al., 2007). The optimum conditions for wheat blast development include a temperature range between 25 and 30°C and spike surface wetness between 25 and 40 h (based on controlled conditions) (Cardoso et al., 2008). Under conducive field conditions, the fungus can kill up to 100% of susceptible wheat spikes in a period of 2.5–3 weeks (Gongora-Canul et al., 2020).

Since 1985, when wheat spike blast was first detected, intense efforts have been undertaken to identify resistance (Igarashi et al., 1986; Urashima et al., 2004; Prestes et al., 2007; Cruz et al., 2016b; Ceresini et al., 2019; Cruppe et al., 2020). Recently, two new genes, Rmg8 and RmgGR119, were found to generate resistance to wheat blast (Wang et al., 2018). However, the only currently effective resistance provided by the 2N^V^S translocation from *Aegilops ventricosa* (Tausch) confers useful yet partial and environment and/or genetic background-dependent resistance to wheat blast (Cruz et al., 2016a; Valent, 2016; Cruppe et al., 2019, 2020). Obtaining tissue samples from phenotyped wheat entries and testing for the presence or absence of the 2N^V^S segment is relatively easy and routine (Cruz et al., 2016b; Yasuhara-Bell et al., 2018; Cruppe et al., 2019). Although there is evidence that 2N^V^S-based resistance may be overcome, additional sources of wheat spike blast resistance should be identified (Cruz et al., 2016b; Cruppe et al., 2019, 2020; Juliana et al., 2020). Thus, there is a continued need to find new sources of resistance to wheat blast.

Plant disease estimations, or phytopathometry, refer to the measurement and quantification of plant disease severity (DS)or incidence that is essential when studying and analyzing diseases at organ, plant, or population levels (Large, 1966; Bock et al., 2010). Plant disease estimations by human raters are the standard method used for plant disease phenotyping. Humans are trained to perform visual disease evaluations of incidence and severity, and their reliability can be improved with experience. These estimations are helpful, but they are subjective evaluations that can introduce variability and can be time-consuming and labor-intensive (Nutter et al., 1993; Madden et al., 2007; Bock et al., 2010, 2020). Due to issues associated with an agreement in data acquisition, inter-rater agreement among other statistical tests can be used to compare the consensus or agreement between estimations of raters of DS (Nutter et al., 1993; Madden et al., 2007; Bock et al., 2010, 2020). These agreement analyses are relevant in plant pathology and plant breeding since inaccurate disease estimations can cause imprecision and unreliability leading to incorrect conclusions (Chiang et al., 2016; Singh et al., 2021).

A bottleneck in the identification of novel sources of resistance is measuring disease intensity (i.e., plant disease phenotyping), which is considered a limiting factor in the assessment of genotype performance in plant breeding programs (Mahlein, 2015; Shakoor et al., 2017). Therefore, innovative and transformative solutions for the quantification of plant disease symptoms at the individual and host population levels are needed (Camargo and Smith, 2009; Kumar et al., 2020). Implementation of advanced computer vision and machine learning techniques could reduce the phenotyping bottleneck during breeding and enhance the understanding of genotype–phenotype relationships (Fiorani and Schurr, 2013; Kruse et al., 2014; Shakoor et al., 2017; Yang et al., 2020; Singh et al., 2021).

Computer vision, machine learning, and deep learning methods have recently been adapted to agriculture due to increased knowledge of algorithms and model capabilities that can learn and make predictions from images Red Green Blue (RGB), multispectral, or hyperspectral (Barbedo, 2016; Kersting et al., 2016; Mahlein et al., 2018). There are two ways in which these models are trained, one is supervised learning, which depends on an annotated dataset, and another is unsupervised learning, which does not rely on annotations (Mahlein et al., 2018). The most frequently used deep learning methods are the Convolutional Neural Networks (CNN). The CNN is characterized by high-accuracy metrics for image recognition and image segmentation. Recent studies have further enhanced the scope of a deep-learning-based approach for classifying, identifying, and quantifying plant diseases (Mahlein et al., 2018; Singh et al., 2018; Barbedo, 2019).

A variety of CNN classification models are available for plant diseases. These include models for bacterial pustule (*Xanthomonas axonopodis pv. glycines*), sudden death syndrome (SDS, *Fusarium virguliforme*), Septoria brown spot (*Septoria glycines*), bacterial blight (*Pseudomonas savastanoi pv. glycinea*), and several abiotic stresses in soybean (Ghosal et al., 2018). In tomato (*Solanum lycopersicum*), deep-learning models were developed with and without pre-training models with images from nine leaf tomato diseases from the website www.PlantVillage.org, obtaining better performance using pre-training models (Brahimi et al., 2018). A total of 54,306 leaf images from several crops with 26 diseases were obtained from PlantVillage.org and trained using AlexNet and GoogleLeNet pre-trained models with a leaf-segmented dataset, obtaining an accuracy of 99.35% (Mohanty et al., 2016). On wheat, an in-field automatic diagnosis system for powdery mildew (*Blumeria graminis f. sp. tritici*), smut (*Urocystis agropyri*), leaf blotch (*Septoria tritici*), black chaff (*Xanthomonas campestris pv. undulosa)*, stripe rust (*Puccinia striiformis f. sp. tritici*), and leaf rust (*Puccinia recondita f. sp. tritici*) were developed using deep-learning, and multiple instances–learning techniques from the Wheat Disease Database 2017 (Lu et al., 2017). Although this database is a significant contribution to wheat disease identification based on images, aspects regarding the reliability of the labeler may be compromised (Lobet, 2017). It is appropriate that detection and quantification studies of plant disease provide evidence of (“true”) estimation agreement analysis before using the labeled images as a dataset for training deep-learning models. Currently, phenotyping of wheat spike blast DS relies on a visual estimation made by humans (Cruz et al., 2016a). We hypothesized that deep CNN models can be trained for wheat spike blast severity image classification under a controlled environment. To test this hypothesis, we focused on the following objectives:

- Evaluate the agreement in data acquisition of the human rater who collected and classified datasets.
- Develop an accurate deep CNN model to detect and classify wheat spike blast symptoms in three severity categories.

## Materials and Methods

### Ethics

A written informed consent was obtained from the individual for the publication of any potentially identifiable images or data included in this article.

### Plant Cultivation and Genetic Materials

Two experiments were conducted under controlled conditions in a growth room at the Asociación de Productores de Oleaginosas y Trigo (ANAPO) research facility in Santa Cruz de la Sierra, Bolivia. Wheat cultivars were planted in pots of 15 cm diameter, filled with vermicast:silt (3:1 [v/v]), and grown at 18−25°C, 14 h light/10 h dark photoperiod, and 50–60% relative humidity. Plants were fertilized, and insecticides were sprayed when needed. Plants were arranged in a randomized complete block design with wheat cultivars having various levels of resistance to MoT, two inoculation levels (inoculated and non-inoculated), and four replicates. Wheat cultivars with a range of sensitivity to the wheat blast were used for the experiments. Experiment one included Bobwhite and South American spring cultivars Atlax, BR-18, Motacú, Urubó, AN-120, Sossego, and San Pablo and for experiment two the cultivars included BR-18, San Pablo, Bobwhite, and Atlax (Baldelomar et al., 2015; Fernández-Campos et al., 2020).

### Inoculation

**Figure 1:**
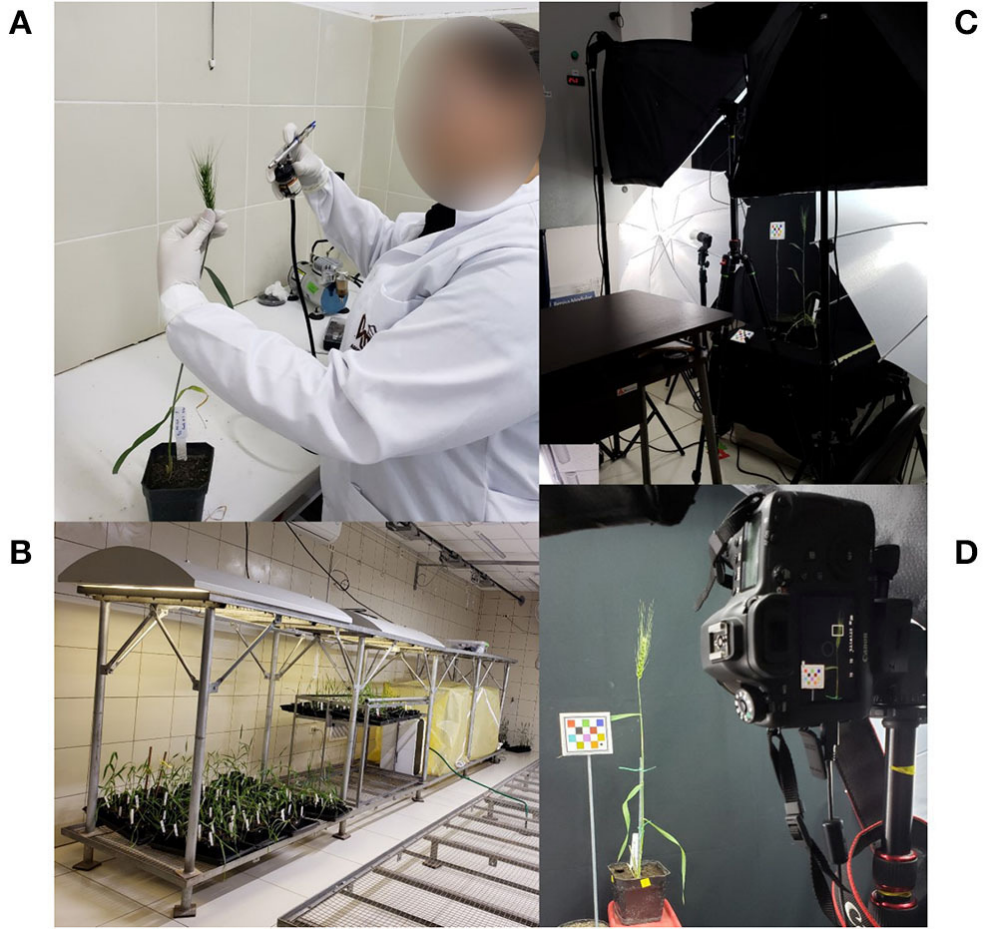
Wheat blast image collection flow process: **(A)** *Magnaphorthe oryzae* pathotype *Triticum* inoculation, **(B)** After inoculation, plants were moved to the yellow dew chamber that provided optimal conditions for fungal infection for 48 h, later transfer to the black trays, **(C)** wheat spike imaging set up, and **(D)** an image was captured perpendicular to the spike.

Plants were inoculated at the growth-stage Feekes 10.5, when the spike had completely emerged, with MoT isolate 008-C (Figure 1A), according to a modified inoculation protocol previously published (Cruz et al., 2016a). A conidial suspension was adjusted to 20,000 spores/ml, and each spike received 1 ml of the spore suspension. Immediately after the spikes were sprayed with the MoT inoculum, plants were moved to a dew chamber (Figure 1B) to induce MoT infection (i.e., 24–26°C, 95–98% RH, and 14 h light photoperiod). Forty-eight hours after inoculation, plants were removed from the dew chamber and left under controlled environment room conditions [(24–26°C and relative humidity of 50–60%), until day 19 after inoculation; Figure 1B].

### Data Collection, DS, and Disease Measurements

Following phytopathometry terminology, we used the term “estimate” for visual disease estimations made by humans and the term “measurement” for estimations made by image analysis (Bock et al., 2010; Gongora-Canul et al., 2020). Visual estimate of DS was obtained by observing the disease area covered in the spike and assigned a corresponding severity value from 0 to 100%. In this study, image analysis disease measurements were achieved by manually measuring spike disease area (pixels) using RGB color threshold segmentation with the image analysis software Fiji ImageJ v.1.52a (Schindelin et al., 2012; Sibiya and Sumbwanyambe, 2019). First, the measurement of the total spike area was obtained, then the diseased area was measured. Finally, the percentage of diseased severity (DS) of the individual spike was calculated (Equation 1), where *A*_*Diseased*_ is the proportion of the area of spike that is diseased divided by the total area of the spike *A*_*Total*_ (See Video 1 in Supplementary Material).

(1) $$\begin{array}{l} DS=\;\frac{A_{Diseased}}{A_{Total}}\;\times 100 \end{array}$$

Visual estimations of wheat spike blast symptoms were taken seven times after inoculation in each experiment. In experiment one, visual estimations and images were collected 4, 6, 9, 12, 14, 16, and 19 days after inoculation (DAI) and in experiment two, 0, 5, 7, 10, 12, 14, and 19 DAI. Each spike side (four sides total) was visually estimated for DS by Rater 1 (a plant pathologist with experience on wheat blast, rice blast, and other diseases). Simultaneously, an image from each spike side was captured perpendicular to the spike with a distance of 50 cm approximately with a DSLR EOS 6D Canon camera (Canon Inc., Tokyo, Japan) (Figure 1D) using a photography studio set up with umbrellas, lights, and screens (Neewer 2.6 m × 3 m/8.5 ft × 10 ft Background Support System and 800 W 5,500 K Umbrellas Softbox Continuous Lighting Kit for Photo Studio Product) that helped create a uniform light and smooth environment (Figure 1C).

### DS Categories

**Figure 2:**
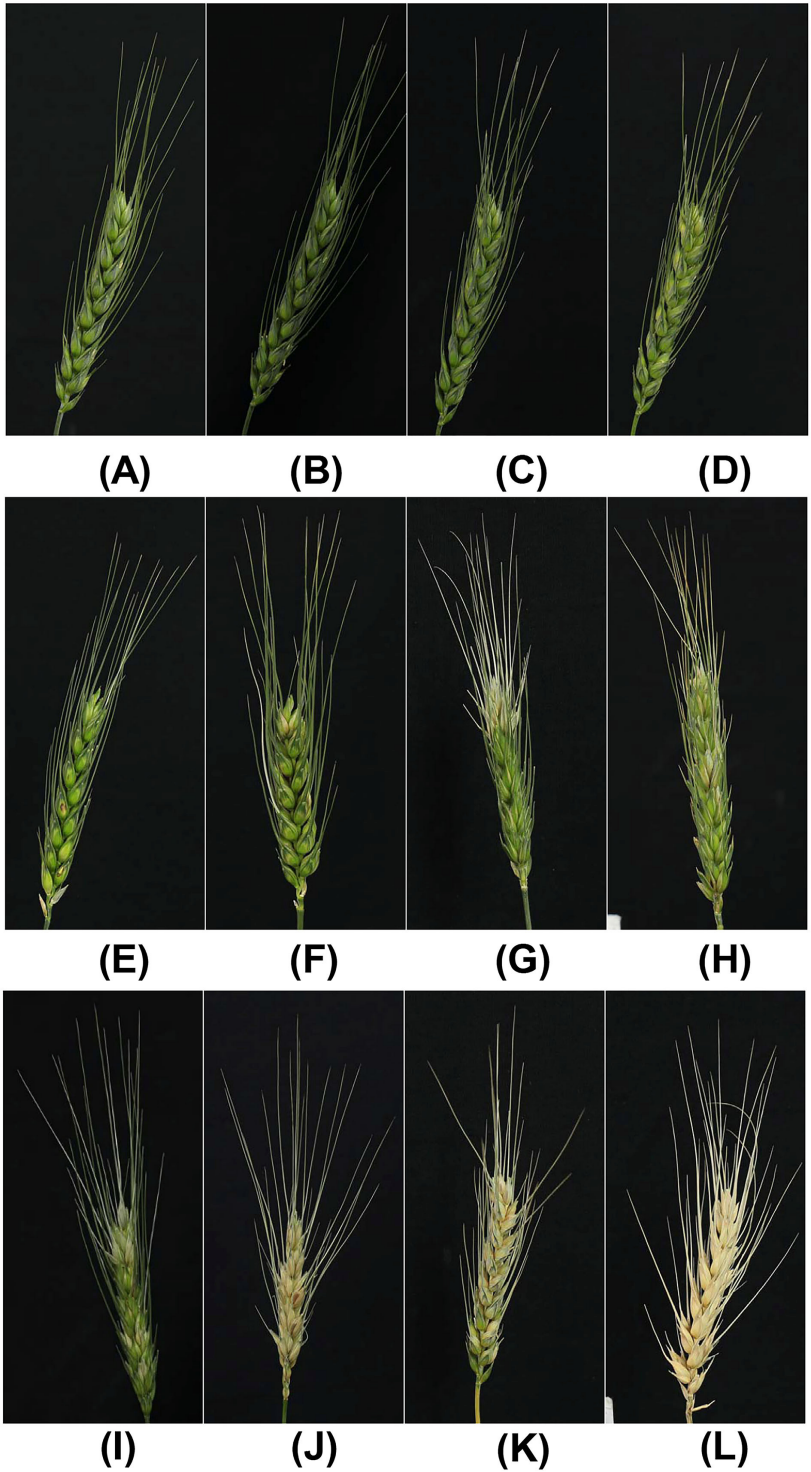
Examples of images per category: **(A–D)** healthy wheat spikes no disease (0% severity, Category 1); **(E–H)** spikes with moderate severity (0.1–20%, Category 2); and **(I–L)** spikes with high severity (20.1–100%, Category 3).

The total spike disease estimations of Rater 1 paired with the corresponding image were converted to a three-category scale according to the amount of severity that served to fed training and testing dataset of CNN model. The category selection was based on wheat blast results from published work conducted over the last decade (Baldelomar et al., 2015; Cruz et al., 2016b; Vales et al., 2018; Cruppe et al., 2020; Fernández-Campos et al., 2020). Category 1 (healthy spikes) was used as a baseline (i.e., negative control or fully immune). Category 2 showed 0.1–20% severity (resistant and moderately resistant/low levels of symptoms) corresponding to the selected putative population for successive trials under variable conditions (controlled environment or field). Category 3 showed 20.1–100% severity (moderately susceptible and susceptible/intermediate and high levels of symptoms) corresponding to the plant population that will not be selected for successive trials because of the high potential to be or to become susceptible to the disease studied (Figure 2).

### Testing Reliability of Rater 1: Inter-rater Analysis of Wheat Spike Blast Severity Estimations

Rater 1 played a critical role in estimating DS and classifying into categories of all the images belonging to Dataset 1 and Dataset 2 (Datasets are described in the section, generation of data sets according to wheat spike physiological changes). Therefore, an inter-rater analysis was needed to determine the reliability of visual estimations of Rater 1. Inter-rater agreement assesses the degree of agreement between two and more raters who obtain independent ratings about the characteristics of a set of subjects. Subjects of interest include people, things, or events that are rated (Madden et al., 2007; Everitt and Anders, 2010; Bock et al., 2020).

To determine the agreement of disease estimations of Rater 1, we performed an inter-rater analysis including a second-rater, and ImageJ was used as an image analysis software baseline. Rater 2, is a plant pathologist and expert in the wheat blast. ImageJ is an image analysis software used to measure plant diseases from images.

We used the power analysis Wilcoxon signed-rank test to determine the sample size for the inter-rater agreement studies of the two training datasets. The test consisted of DS estimations or measurements of 31 and 29 images from the CNN training Dataset 1 and training Dataset 2, respectively. From now on, the 31 images selected from Dataset 1 will be called sample Dataset 1 and the 29 images from Dataset 2 will be referred to as sample Dataset 2. Rater 2, who is an experienced researcher with more than 4 years of working with the wheat blast disease, visually estimated DS from the sample Dataset 1 and Dataset 2. Additionally, disease measurements were obtained from the sample Dataset 1 and the sample Dataset 2 using ImageJ software as indicated above. Ultimately, the DS results of visual disease estimations of human raters and ImageJ measurements were compared. The estimated and measured DS values from both samples were analyzed for inter-rater agreement in two scenarios, one with a scale of 0–100% DS (continuous data), and the other with the images divided into three categories of DS (ordinal data). We, therefore, computed Lin's Concordance Coefficient, Fleiss kappa, and weighted kappa statistics.

The Lin's concordance coefficient (ρc or CCC) is used to estimate the accuracy_1_ between two raters using continuous data. From the analysis, we obtained the estimation of accuracy_1_, precision_1_, and bias of the disease estimations and disease measurements between the two raters (Lin, 1989; Madden et al., 2007; Bock et al., 2010). For accuracy_1_ (ρc) and precision_1_ (r), values range from 0 to 1; values close to 1 indicate high accuracy_1_ and precision_1_. Bias (Cb) ranges from 0 to 1, and values close to 1 indicate less bias (Nita et al., 2003). Lin's concordance analysis was performed by using PROG REG ALL procedure on SAS v.9.4 (Cary, NC), based on the macro developed by Lawrence Lin and verified by Min Yang (Lin et al., 2002).

To determine the degree of association between the estimation of categorical information provided by the two raters (inter-rater agreement), the weighted kappa statistics were computed (Chmura, 1992; Graham and Jackson, 1993; Nelson and Edwards, 2015). The Fleiss kappa coefficient was used to compare the agreement of categorical information among all raters, (i.e., Rater 1, Rater 2, and ImageJ) (Fleiss et al., 2003). The values of both the weighted kappa and Fleiss kappa coefficients range from 0 to 1. Values from 0.5 to 1 indicate that the agreement is better than what is expected by chance (Nelson and Edwards, 2015; Tang et al., 2015; Mitani et al., 2017; Gamer et al., 2019). The Fleiss kappa statistics and weighted kappa were computed with the *irr* package of the R software (Team, 2017).

### Generation of Datasets According to Wheat Spike Physiological Changes

Wheat was inoculated at the growth-stage Feekes 10.5 (spike completely emerged) of the host plant. Approximately every 2 days after the inoculation, the spike images were collected to capture the changes developed. Indirectly, progressive physiological changes in spikes were recorded, as maturing begins at wheat growth-stage Feekes10.5.4 (kernels watery ripe) and continues through the growth-stage Feekes 11.4 (mature kernels) (Large, 1954; Wise et al., 2011). During this period, the kernel hardened, and the green spike lose its color (maturing), which mimic the typically bleached spikes caused by wheat spike blast symptoms.

Two datasets were generated considering the (color) physiological changes that can lead to confusion when training the CNN model. Dataset 1, included maturing and non-matured wheat spikes; and Dataset 2 included only non-matured spikes (data available at: https://purr.purdue.edu/publications/3772/1). The proposed CNN model was trained using the two datasets. Each dataset was randomly separated into the training and testing datasets. The CNN model automatically extracted the features of each image in the training dataset to learn a good classifier, whereas the testing dataset was used to evaluate the performance of the trained CNN model. In general, an unseen dataset was applied to evaluate the CNN model to ensure that the model was not under-fitting or over-fitting. In this research, 80% of the images were categorized and used as the training set, and the remaining 20% as the testing set. Table 1 lists the original distribution of the number of images in Dataset 1 and Dataset 2. Although Category 3 covers a large variability, it does not mean the number of the data in Category 3 is larger than the other two categories. The number of images in each category was extremely imbalanced and using them indiscriminately could have resulted in a biased model. Fortunately, there are several viable methods to cope with the disproportionate training data in each category.

**Table 1:** Training and testing data distribution and the number of images used in Dataset 1 and Dataset 2.

**Sets**	**Category 1**	**Category 2**	**Category 3**
**Dataset 1 (Maturing and non-matured spikes)**			
Training	1,595	640	402
Augmented training	1,595	1,920	1,608
Testing	381	178	110
**Dataset 2 (Non-matured spikes only)**			
Training	1,430	386	307
Augmented training	1,430	1,544	1,535
Testing	327	120	90

*Category 1: 0% severity, Category 2: 0.1–20% severity, Category 3: 20.1–100% severity*.

Data augmentation is a common technique providing a viable solution to data shortage issues by adding copies of original images with modification or noise (Boulent et al., 2019). Data augmentation was used in this study to balance the number of images in each category. In this study, images were randomly flipped horizontally and vertically in order to increase the number of images in Categories 2 and 3. Thus, for Dataset 1, training data were triplicated in Category 2 and quadrupled in Category 3 (Table 1). For Dataset 2, training data were quadrupled in Category 2 and quintupled in Category 3 (Table 1).

### Deep CNN Model

In recent years, the feasibility of using artificial intelligence, in particular deep learning, has been expanded into a variety of applications (Atha and Jahanshahi, 2018; Chen and Jahanshahi, 2018; Kumar et al., 2018; Wu and Jahanshahi, 2019). Deep learning is a subset of machine learning that enables computers to automatically extract features from a huge amount of data and learn to classify data.

In this study, wheat spike blast symptoms were automatically detected and classified into three severity categories using a pre-trained CNN model. This model may be more efficient than classifying images visually. To obtain a general and reliable CNN model, the network needed to be trained using a large labeled training dataset. The performance of the CNN model is highly dependent on the number and quality of the training data. However, it was hard to collect a wheat blast dataset having a million images in a short time. The performance CNN model can easily lead to under- or over-fitting due to the lack of a large dataset for training. To address this issue, transfer learning was used as a practical solution where a network was trained using a typically different larger dataset such as ImageNet. A major advantage of using transfer learning is that it can adapt the parameters trained from an abundant number of images. Transfer learning starts with a pre-trained model, e.g., VGG16 model, and replaces the fully-connected (FC) layers of the model with new FC layers. A network trained on the ImageNet dataset was used to initialize the network parameters, and the whole network was fine-tuned since the nature of our dataset was very different from the ImageNet dataset. In this study, an FC layer that consisted of three nodes, representing three categories, were appended to the end of the network. A residual neural network architecture (ResNet101), a CNN model with 101 layers with recurrent connection trained on ImageNet data (He et al., 2015, 2016), was selected as the pre-trained model. Furthermore, as shown in Table 1, it was extremely difficult to obtain a large number of images in each category. An unbalanced dataset can result in a biased CNN model. To address this issue in the dataset, the loss function, which was used to optimize the parameter in a neural network, was transformed into a weighted loss function (Equation 2) by assigning individual weights to each category. Equation (2) defines the cross-entropy loss function in the CNN model, where ω_category_ is the assigned weight to each of the categories, the first term in Equation (2) is a negative log-likelihood loss, and the second term in Equation (2) is log-softmax. Four cases of study were tested with an individual weight set to the loss functions assigned to different categories. In the experiments, “cases” refer to specific combinations of weight loss functions for each of the three DS categories (Table 2). Case 1 was the non-weight set [1, 1, 1], with all categories sharing the same class weight. Case 2 used [1, 10, 1] class weights in the loss function, meaning that the highest weight was for Category 2, which includes plants at early disease stages and low levels of disease symptoms. Case 3 used [2, 5, 1] class weights in the loss function, meaning that the higher weight was assigned to Categories 1 (no symptoms) and 2 (early stages and low levels of disease symptoms). Case 4 had class weights [2, 1, 1] in the loss function, assigning a higher weight to category 1 (no symptoms) (Table 2).

(2) $$\begin{array}{l} loss\left(x,\;category\right)=-\omega_{{\text{category }}^{*}}\log\frac{e^{x_{\text{category}}}}{\sum_{j=1}^{N}e^{x_{j}}}\\ \;=-\omega_{\text{category }}\left(-x_{\text{category }+\;}\text{log }\left(\underset{\text{j}}{\sum }\text{exp}\left(x_{j}\right)\right)\right) \end{array}$$

The network was trained for 15 epochs using a stochastic gradient descent optimizer (Bottou, 2010), a learning rate of 0.0001 was used, and the batch size was 16. Additionally, 5-folds cross-validation was applied to the training process. The training took place on a Linux server with Ubuntu 14.04. The server included two Intel Xeon E5-2620 v4 CPUs, 256-GB DDR4 memories, and four NVIDIA Titan X Pascal GPUs. Pytorch (Paszke et al., 2017) was used to implement the CNN.

**Table 2:** Two datasets trained the CNN model with four cases of the study through different weights in loss functions for each category.

	**Values of weighted loss function per category [1, 2, 3]**	
**Model**	**Dataset 1 (Maturing and non-matured spikes)**	**Dataset 2 (Non-matured spikes)**
Case 1	[1, 1, 1]	[1, 1, 1]
Case 2	[1, 10, 1]	[1, 10, 1]
Case 3	[2, 5, 1]	[2, 5, 1]
Case 4	[2, 1, 1]	[2, 1, 1]

*[Category 1: 0% severity, Category 2: 0.1–20% severity, Category 3: 20.1–100% severity]*.

### Model Performance Evaluation

The performance of the CNN model was evaluated *via* the classified results of the testing dataset. A 3 × 3 confusion matrix was used to describe the prediction result of the model. Each row of the confusion matrix represented the ground truth of the data, and each matrix column corresponded to a predicted category by the CNN model. Thus, the diagonal elements of the matrix, called true positive (TP), were the number of wheat images correctly classified into the ground truth. The false positive (FP) for each Category was the sum of all errors in that column. For example, the FP of Category 1 was the number of Category 2 and Category 3 severities that were incorrectly classified as Category 1. Based on the confusion matrix, additional evaluation metrics were calculated.

Accuracy_2_ was defined as the total number of TP among three categories divided by the total number of the predictions. Precision_2_ was defined as the total number of the TP instances divided by the total number of predicted positive examples, which was the summation of TP and FP instances in the binary classification task (Equation 3). Similarly, the precision_2_ of the multi-classes task illustrates the number of instances that were correctly predicted given all the predicted labels for a given category. Recall was defined as the TP instance divided by all the positive samples (TP and FN) (Equation 4). F1 score is a single metric that encompasses both precision_2_ and recall (Equation 5). Accuracy_2_, precision_2_, recall, and F1 score metrics ranged from 0 to 1, where higher values indicate the high predictive ability of the model.

(3) $$\begin{array}{l} Precision_{2}=\;\frac{TP}{TP+FP\;} \end{array}$$

(4) $$\begin{array}{l} Recall=\;\frac{TP}{TP+FN} \end{array}$$

(5) $$\begin{array}{l} F_{1}score=2\;\times \;\frac{precision\;\times \;recall}{precision+\;recall\;} \end{array}$$

## Results

### Cultivar Response to Wheat Spike Blast Under Controlled Conditions

The final wheat spike blast severity was at day 19 after inoculation when cultivar Atlax reached 100% average DS, followed by Bobwhite (99.7%), San Pablo (32.9%), BR-18 (8.7%), Motacú (3.7%), AN-120 (3.31%), Urubó (1.9%), and Sossego (0.83%). Wheat spike blast symptoms developed on all tested cultivars, with reactions to MoT infection consistent with previous reports, except for cultivar San Pablo that showed moderate susceptibility (Baldelomar et al., 2015; Cruz et al., 2016b; Cruppe et al., 2020; Fernández-Campos et al., 2020; Gongora-Canul et al., 2020). Cultivar Atlax exhibited the highest DS of all the cultivars and had a high level of susceptibility to wheat spike blast.

### Inter-rater Agreement Analysis

**Figure 3:**
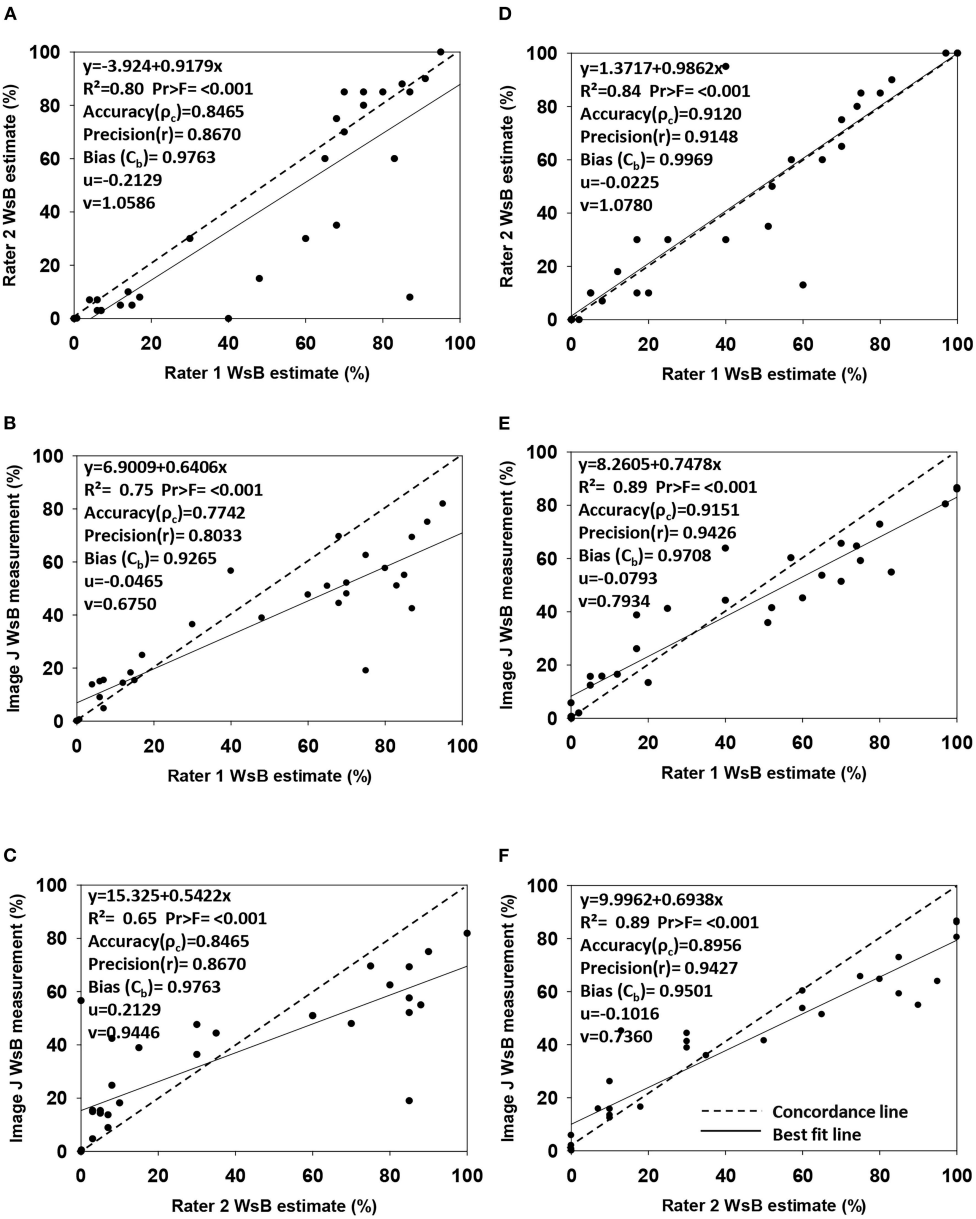
Regression analysis of wheat spike blast DS estimations made by Rater 1 (responsible to estimate the severity of total image dataset) *vs*. Rater 2 (expert in wheat blast) and ImageJ DS measurements (image analysis software). Graphs show accuracy (ρ_c_), precision(r), bias (Cb), scale shift (ν), and location shift (u) for wheat spike blast continuous Dataset 1 **(A–C)** (*n* = 31 images) and Dataset 2 **(D–F)** (*n* = 29 images). **(A)** Disease estimation comparison from images Dataset 1 between Rater 1 and Rater 2. **(B)** Disease estimation and disease measurement comparison from images Dataset 1 between Rater 1 and ImageJ. **(C)** Disease estimation and disease measurement comparison from images Dataset 1 between Rater 2 and ImageJ. **(D)** Disease estimation comparison from images Dataset 2 between Rater 1 and Rater 2. **(E)** Disease estimation and disease measurement comparison from images Dataset 2 between Rater 1 and ImageJ. **(F)** Disease estimation and disease measurement comparison from images Dataset 2 between Rater 2 and ImageJ.

The Lin's concordance correlation analysis showed a high accuracy_1_ (ρc = 0.89–0.91), high precision_1_ (r = 0.91–0.94), and less bias (Cb = 0.95–0.99) in the sample Dataset 2 than in the sample Dataset 1 (ρc = 0.77–0.85, precision_1_ r = 0.80–0.87, and bias Cb = 0.93–0.98) (Figure 3). In the sample Dataset 1, the highest accuracy_1_ was between Rater 1 and Rater 2 (ρc = 0.85) and between Rater 1 and ImageJ (ρc = 0.85). In the sample Dataset 2, the highest accuracy_1_ value was between Rater 1 and ImageJ (ρc = 0.92), followed by between Rater 1 and Rater 2 (ρc = 0.91). In both sample datasets, strong accuracy_1_, high precision_1_, and low bias involved Rater 1, providing evidence that ratings of disease based on continuous data were done correctly for further classification of the images into categories for model training.

The weighted kappa statistics (κ), used to quantify inter-rater agreement, were higher in the sample Dataset 1 than in the sample Dataset 2, with κ = 0.72–0.88 (*p* < 0.01) and κ = 0.78–0.85 (*p* < 0.01), respectively (Table 3). In the sample Dataset 1, the highest agreement occurred between Rater 1 and ImageJ (κ = 0.88), and in the sample Dataset 2, the highest agreement was between Rater 1 and Rater 2 (κ = 0.85). In both sample datasets, the substantial agreement involved the ground truth (Rater 1), providing evidence that ratings were done correctly for further classification of the images into categories for model training.

**Table 3:** Values of weighted Kappa (κ) analysis for inter-rater agreement between raters and ImageJ in Dataset 1 (maturing and non-matured spikes) and Dataset 2 (non-matured spikes) of wheat spike blast under controlled environment.

	**Dataset 1**		**Dataset 2**	
**Categories**	**κ**	**z**	**κ**	**z**
Rater 1^x^ *vs*. ImageJ^z^	0.882^**^	4.93	0.822^**^	4.45
Rater 2^y^ *vs*. ImageJ	0.727^**^	4.13	0.776^**^	4.32
Rater 1 *vs*. Rater 2	0.747^**^	4.32	0.849^**^	4.65

** *p < 0.01*.

x *Rater 1: Responsible to estimate the severity of the total image dataset*.

y *Rater 2: Expert in the wheat blast*.

z *ImageJ: Image analysis software*.

The Fleiss kappa coefficient (Fκ), which compared the association of ordinal categorical information of two or more raters, showed an Fκ = 0.771 (*n* = 31, z = 9.26, *p* < 0.001) for the sample Dataset 1 and 0.697 (*n* = 29, z = 8.1, *p* < 0.001) for the sample Dataset 2, indicating substantial agreement among the human raters and ImageJ in both datasets. However, the sample Dataset 1 possessed a higher Fleiss kappa coefficient index than the sample Dataset 2, both presented substantial agreement between the rates and ImageJ. Yet, the evidence supported the fact that the three raters correctly estimated the amount of the disease from the same image.

### Deep CNNs Model Performance

To train the proposed CNN model, two different datasets were used. As mentioned above in the section *Generation of Datasets According to Wheat Spike Physiological Changes*, testing reliability of Rater 1, Dataset 1 included matured and non-matured wheat spikes and Dataset 2 included only non-matured spikes (Table 1). Four cases applied different weight set of loss functions in both Datasets (Table 2, Supplementary Figures 1, 2). The performance of the CNN model was evaluated *via* the classified result of the testing data.

The testing accuracy_2_ of the model trained with Dataset 1 was 90.1% in Case 1, 90.4% in Case 2, 90.0% in Case 3, and 87.7% in Case 4. The testing accuracy_2_ of Dataset 2 was 98.4% in Case 1, 93.9% in Case 2, 95.0% in Case 3, and 94.2% in Case 4. Dataset 2 presented higher accuracy_2_ values compared to Dataset 1, suggesting that the model was accurate. However, it was not sufficient to claim that the model was reliable based on accuracy_2_ alone since the dataset in this study was unbalanced. In addition to accuracy_2_, other metrics can help evaluate the performance of the CNN model, such as precision_2_, recall, and F_1_ score.

**Figure 4:**
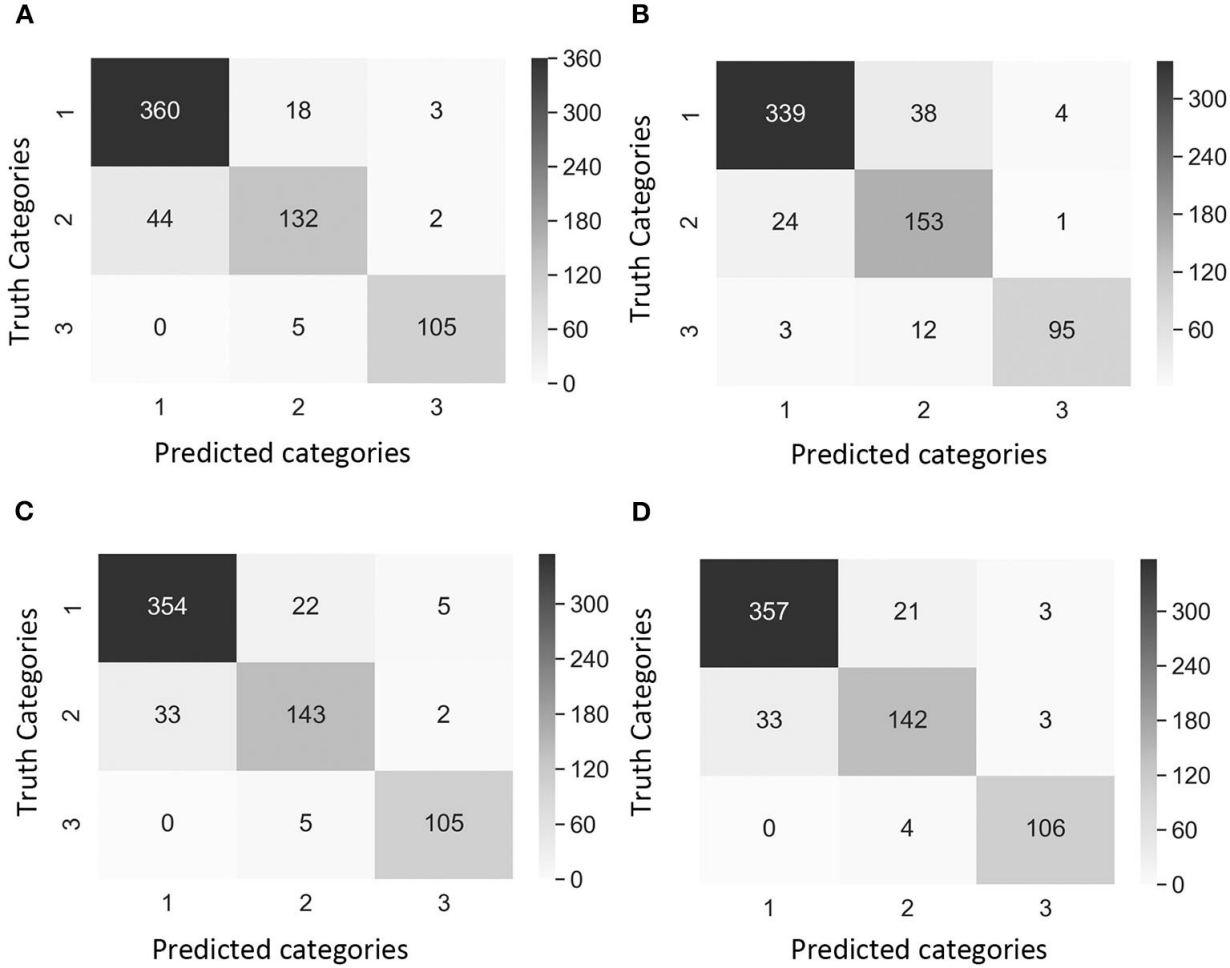
Confusion matrix of the images of Dataset 1 (non-matured spikes only) showing “true” categories by Rater 1 (y-axis) and predicted categories by the CNN model (x-axis). Category 1: contained images with 0% severity, Category 2: 0.1–20% severity, Category 3: 20.1–100% severity. The cases of study presented different weights in the loss function [weight in Category 1, weight in Category 2, weight in Category 3]. **(A)** Case 1 [1, 1, 1], **(B)** Case 2 [1, 10, 1], **(C)** Case 3 [2, 5, 1], and **(D)** Case 4 [2, 1, 1]. Values and color intensity represent number of images.

Precision_2_ indicates the ability to correctly classify an instance in all predicted positive instances. The focus was on the performance of the CNN model in Category 2 as this was the category that breeders and pathologists will concentrate on for breeding purposes. Dataset 1 Case 2 showed the lowest precision_2_ (75.4%) among all cases values (Table 4). Moreover, the confusion matrix of Dataset 1 Case 2 showed that the model misclassified 38 images of Category 1 (no symptoms) as Category 2 (early disease stages and low levels of disease symptoms), which was the highest number of wrongly classified images among all the cases (Figure 4B). This suggested that the class weight of Category 2 might be too high since its misclassified images that belonged to other categories as Category 2. Hence, the class weight combination was modified by lowering the weight in Category 2 and increasing the weight in Category 1 as to not overemphasize the impact from Category 2. Precision_2_ of Category 2 significantly increased from 75.4% in Case 2 to 84.1% in Case 3, and to 85.0% in Case 4 (Table 4). In Case 2, precision_2_ of Category 2 significantly increased from 75.4% in Dataset 1 to 90.2% in Dataset 2 (Table 4). Precision_2_ of Category 2 significantly increased from 90.2% in Case 2 to 92.7% in Case 3 and from 90.2% in Case 2 to 94.1% in Case 4 (Table 4).

**Table 4:** Classification performance of the CNN model when classifying the testing set of Dataset 1 (maturing and non-matured spikes) and Dataset 2 (non-matured spikes) in the cases of the study presented different weights in the loss function [weight in Category 1, weight in Category 2, weight in Category 3].

		**Dataset 1**			**Dataset 2**		
**Model**	**Performance Index**	**Category 1**	**Category 2**	**Category 3**	**Category 1**	**Category 2**	**Category 3**
Case 1^(A)^	Precision	0.891	0.852	0.955	0.923	0.918	0.967
	Recall	0.945	0.742	0.955	0.985	0.750	0.967
	F-1 score	0.917	0.793	0.955	0.953	0.826	0.967
Case 2^(B)^	Precision	0.926	0.754	0.950	0.952	0.902	0.936
	Recall	0.890	0.860	0.864	0.963	0.842	0.978
	F-1 score	0.908	0.803	0.905	0.957	0.871	0.957
Case 3^(C)^	Precision	0.915	0.841	0.938	0.953	0.927	0.967
	Recall	0.929	0.803	0.955	0.985	0.842	0.967
	F-1 score	0.922	0.822	0.946	0.968	0.882	0.967
Case 4^(D)^	Precision	0.915	0.850	0.946	0.942	0.941	0.946
	Recall	0.937	0.798	0.964	0.991	0.792	0.967
	F-1 score	0.926	0.823	0.955	0.966	0.860	0.956

(A) *Case 1 [1, 1, 1]*,

(B) *Case 2 [1, 10, 1]*,

(C) *Case 3 [2, 5, 1], and*

(D) *Case 4 [2, 1, 1]*.

*The performance measures per class considered were precision, recall, and F_1_ score*.

The recall metric for evaluating the CNN model that indicates the ability to correctly recognize a category was also used. In datasets 1 and 2, the recall of Category 2 was the lowest, illustrating the challenge of the model to classify images of Category 2 (early disease stages and low levels of disease symptoms) (Table 4). The highest recall of Dataset 1 Category 2 was 86.0% in Case 2, and the lowest was 74.2% in Case 1 (Table 4). This was expected given that Case 2 had a higher weight in the loss function of Category 2 compared to Case 1 (non-weighted loss function). In Case 2, Dataset 1, the recall values were similar among the three categories (Table 4). In Dataset 2 Category 2, the lowest recall was 75.0% in Case 1, and the highest recall was 84.2% in cases 2 and 3 (Table 4). The model in these two cases had the highest weight in loss function of Category 2 (early disease stages and low levels of disease symptoms).

F_1_ score is a common indicator of the overall performance of the CNN model. In datasets 1 and 2, the F_1_ score of Category 2 was the lowest, reaffirming the difficulty of classifying images of Category 2 by the model (Table 4). The lowest F_1_ score of Dataset 1 Category 2, was 79.3% in Case 1, while the highest was 82% in both Case 3 and Case 4 (Table 4). In Dataset 2 Category 2, the lowest F_1_ score was 82.6% in Case 1, and the highest F_1_ score was 88.2% in Case 3 followed by Case 2 with 87.1% (Table 4).

**Figure 5:**
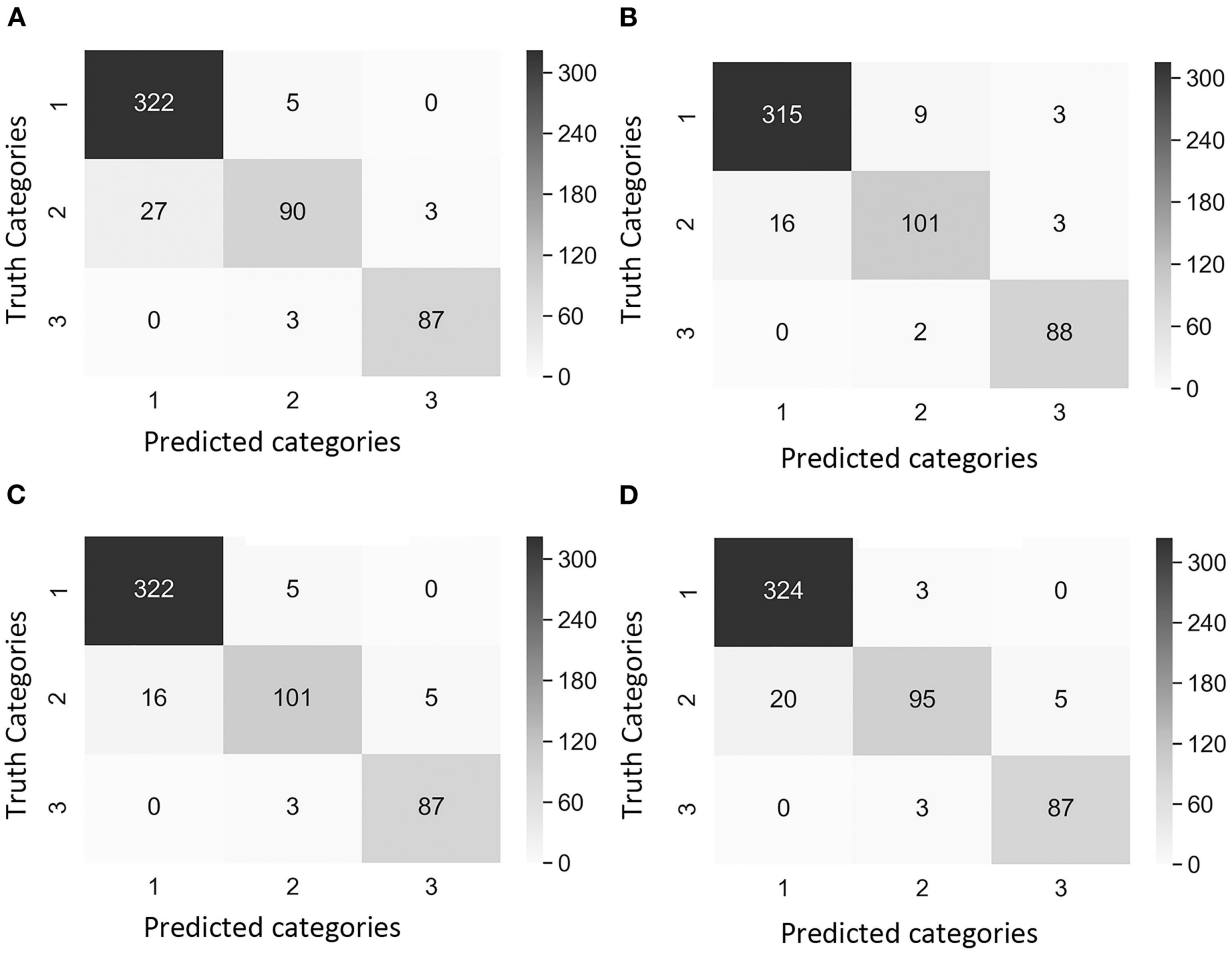
Confusion matrix of the images of Dataset 2 (non-matured spikes only) showing “true” categories by Rater 1 (y-axis) and the predicted categories by the CNN model (x-axis). Category 1: contained images with 0% severity, Category 2: 0.1–20% severity, Category 3: 20.1–100% severity. The cases of study presented different weights in the loss function [weight in Category 1, weight in Category 2, and weight in Category 3]. **(A)** Case 1 [1, 1, 1], **(B)** Case 2 [1, 10, 1], **(C)** Case 3 [2, 5, 1], and **(D)** Case 4 [2, 1, 1]. Values and color intensity represent number of images.

A comparison of outcomes revealed that Category 2 was the most difficult category to classify correctly (Figure 4). This difficulty was attributed to the disease symptoms being barely visible at the early stage of infection, and some wheat spikes in Category 1 were maturing, and their color was similar to that of MoT infected spikes. We observed that the highest number of images exactly classified as Category 2 was obtained with the Case 2 Dataset 1 (Figure 4B). These results suggested that Case 2 was the most appropriate to classify wheat spike blast images in Dataset 1 because it was capable of detecting the infection at an early stage. Even though Case 2 had a slightly lower precision, this is considered the usual trade-off between precision_2_ and recall for disease classification purposes. The recall, precision_2_, and F_1_ score increased after the images of maturing spikes were omitted when training the model with Dataset 2 (Figure 5). The cases 2 and 3 of Dataset 2 presented the highest number of images exactly classified as Category 2 (Figures 5B,C). Cases 2 and 3 were the most appropriate to detect the wheat spike blast in Dataset 2 because the model was capable of detecting the infection in the early stages. Additionally, in all the cases, the model was more stable predicting Category 3, which is relevant because it covers DS from 20.1 to 100%, potentially aiding breeders and pathologists to discern higher levels of susceptibility among cultivars. Although the CNN model misclassified some images of Category 2, it still provided a promising approach to classify the severity of the disease. It demonstrated that the CNN model is potentially a good method for breeders and pathologists.

## Discussion

Wheat blast is spreading worldwide, the identification of durable and broad-spectrum resistance is urgently needed (Valent et al., 2021). There are a few known sources of effective resistance, and therefore it is crucial to identify more genetic resources. Plant disease phenotyping is a bottleneck in the identification of novel sources of resistance. We developed the first deep CNNs model for wheat spike blast phenotyping under controlled environment.

This study results demonstrated that the agreement between disease estimations and disease measurements was more significant than what could have been expected to occur by chance. Rater 1 (a pathologist with expertise in multiple diseases besides blast) consistently obtained the higher kappa coefficient (substantial agreement), higher accuracy, and lower bias in all the performed analyses than disease estimations of an expert (Rater 2) in the wheat blast and the disease measurements of ImageJ software. These results are relevant because Rater 1 estimated the DS and classified the entire image dataset into three categories. Therefore, the agreement analysis supports an accurate classification of the images before they were used to train and test the CNN model. The inter-rater agreement analysis also showed that accuracy, precision, and bias are highly dependent on the nature of the dataset. Dataset 1 included images showing disease symptoms and natural plant physiological changes. However, although Dataset 2 was preferred due to higher concordance, results showed that DS assessments among raters were never perfect.

In the present study, the applicability of CNNs for wheat spike blast severity classification from spring wheat images was investigated. Currently, the CNN approach can classify three severity levels (0%, 0.1–20%, and 20.1–100% severity) and was trained using a reliable wheat spike blast dataset. The advantage of this three categories CNN model is that it detects the infected wheat spike and provides further information on the corresponding blast severity level. It is useful to have such a model to classify different infection levels and identify the resistant cultivars from the susceptible ones. Despite the wheat blast dataset comprising of imbalanced data that could have led to a biased CNN model, two techniques, including data augmentation and weighted loss function, were applied to the training process. The loss function is a function map of the difference between the ground truth and predicted output of the model. The importance of a category with a larger error can be enhanced by assigning a weighted variable in the loss function. The results indicate that the performance of the model has a significant improvement when the weighted loss function is applied. In particular, the model has gained the ability to detect Category 2 using a weighted loss function. These encouraging results demonstrate that the proposed CNN model can distinguish Category 1 and Category 2 even though there is a relatively little difference between both the categories. More significant, the CNN could classify the images of Category 3 with low error, which contained infected spikes with severities higher than 20%.

The results showed that the CNN models trained in both datasets (Fernandez-Campos et al., 2021) presented good performance classifying the wheat spike blast images in the corresponding severity categories. However, the models trained without images of wheat maturing spikes showed higher precision_2_, recall, and F_1_ score when classifying the images than the models trained with maturing and not matured wheat spikes. The performance of the model trained with maturing and non-matured spikes is a critical finding from a biological/physiological point of view. These symptoms on spikes are often reported when wheat has reached the medium milk-to-dough growth stage (Cruz et al., 2016b). The reason why the rating is often stopped at the milk-to-dough stage is that from that point forward, physiological maturity starts to kick in. Our findings will serve to provide future and explicit guidelines to potential users of the preferred model. Users will need to acknowledge the natural wheat maturity process (which alters spike color from green to yellow/white), which can confuse the CNN model. This statement applies when phenotyping for wheat blast or similar diseases with symptoms characterized by spike bleaching [e.g., *Fusarium graminearum* (Fusarium head blight)].

Different software based on image analysis are currently available to measure DS (Lamari, 2002; Vale et al., 2003). We used ImageJ, a free image-processing software, and manually thresholded images to measure wheat spike blast severity. de Melo et al. (2020) indicated the inevitable error when delineating the disease area with image analysis software (Bock et al., 2008). This is a challenge that future research needs to address when disease symptoms are not well-defined.

Researchers could benefit from the proposed approach promising for wheat spike blast severity measurements under controlled environmental conditions. Results are supported by a substantial agreement with “true” data obtained from Rater 1, compared against disease estimations of Rater 2, and disease measurements of ImageJ. In collaboration with data scientists, breeders could pre-select wheat cultivars under controlled environments by automatically analyzing and classifying images using the wheat spike blast CNN model preferably trained with Dataset 2. Next, the breeders can focus on the cultivars that fall into categories 1 and 2, which in general terms, are considered resistant or moderately resistant. This may reduce the high number of cultivars tested under field conditions, accelerating the cultivar screening process. A limitation of the study is that the CNN was trained to classify only images of wheat spike blast (spring wheat) under controlled conditions. Further research is required to improve the generalizability of the CNN model using a greater wheat spike blast dataset consisting of controlled and field images. In addition, the results in this study show an opportunity that could be applied similar to other pathogens.

The next step in this research is to validate the model with other images with a similar background and deploy it in a Web application. This future option might allow breeders and pathologists to submit their images and have the model classify them by categories automatically. As more images of various cultivars infected with different isolates can be added to the dataset, increasing symptom variability, a more refined and robust model can be developed. To our knowledge, this is the first study presenting a deep CNN model trained to detect and classify wheat spike blast symptoms. The model might help in the pre-screening of wheat cultivars against the blast fungus under controlled conditions in the future.

## Data Availability Statement

The raw data supporting the conclusions of this article and corresponding models are available at: https://purr.purdue.edu/projects/wheatspikeblastcnn/publications/3772.

## Author Contributions

MF-C, CDC, MJ, Y-TH, TW, and JJ contributed to the study's conception and design. MF-C and CDC conducted the experiment. MF-C collected data and wrote the first draft of the manuscript. MF-C and CG-C performed the statistical analysis. Y-TH and TW wrote the code for the model. Y-TH wrote sections of the manuscript. CDC, MJ, Y-TH, DT, and CG-C edited the manuscript. All authors approved the submitted version.

## Conflict of Interest

The authors declare that the research was conducted in the absence of any commercial or financial relationships that could be construed as a potential conflict of interest.
