# Supplementary material for: Wheat Spike Blast Image Classification Using Deep Convolutional Neural Networks
Source: Front Plant Sci. 2021 Jun 17;12:673505. doi: 10.3389/fpls.2021.673505 (PMC8248543; doi:10.3389/fpls.2021.673505)
Supplement: Supplementary file 1 [file Data_Sheet_1.pdf]

Supplementary Material

1 Supplementary Data

The CNN code, wheat spike blast image datasets, and a video using ImageJ to measure wheat blast severity are available at: https://purr.purdue.edu/projects/wheatspikeblastcnn/publications/3772.

2 Supplementary Figures

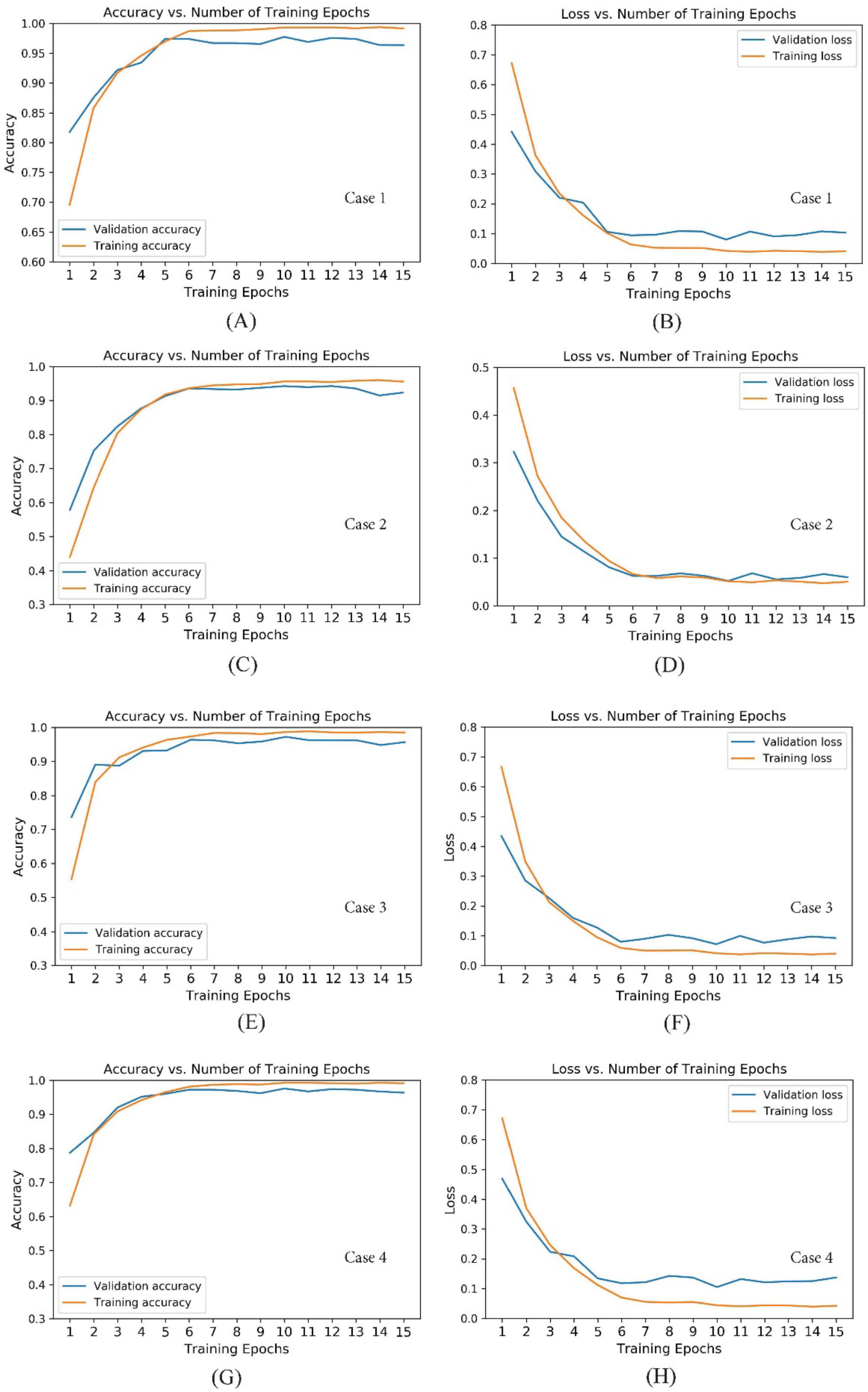

Supplementary Figure 1. The learning curves of the CNN models Case 1 to Case 4 trained on Dataset 1 are shown. The cases of study presented different weights in the loss function, [weight in

Category 1, weight in Category 2, weight in Category 3]. The plots (A) and (B) represent the learning curves of Case 1 [1,1,1], (C) and (D) Case 2 [1,10,1], (E) and (F) Case 3 [2,5,1] and (G) and (H) Case 4 [2,1,1].The left plots show training (orange solid line) and validation (blue solid line) accuracy versus the number of the epoch trained on Dataset 1. The right plots represent the loss versus the number of the epoch trained on Dataset 1.

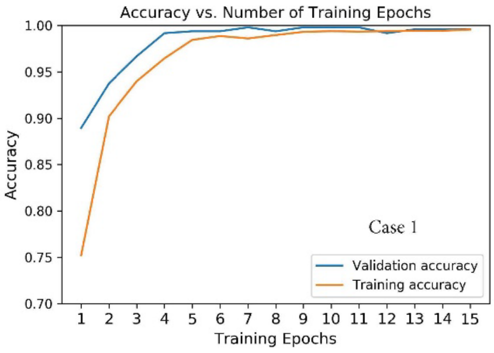
Accuracy vs. Number of Training Epochs
Accuracy
Training Epochs
Case 1
Validation accuracy
Training accuracy

(A)

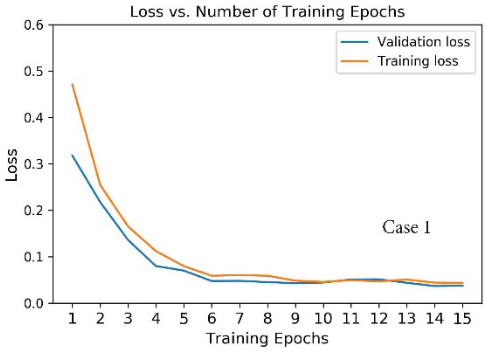
Loss vs. Number of Training Epochs
Loss
Training Epochs
Validation loss
Training loss
Case 1

(B)

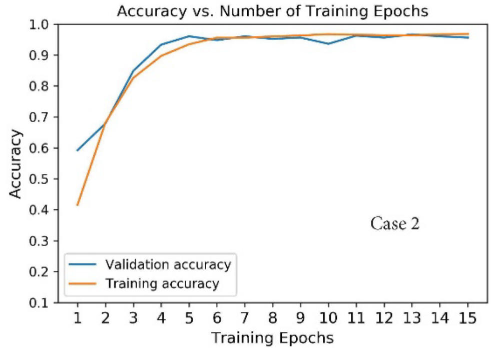
Accuracy vs. Number of Training Epochs
Accuracy
Training Epochs
Case 2
Validation accuracy
Training accuracy

(C)

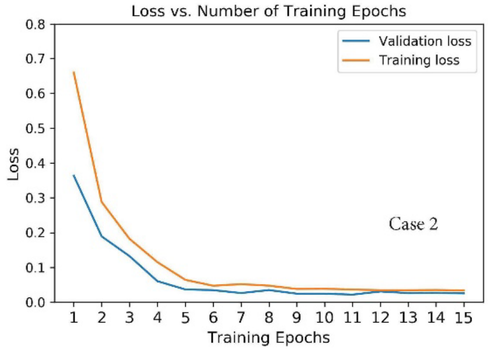
Loss vs. Number of Training Epochs
Loss
Training Epochs
Validation loss
Training loss
Case 2

(D)

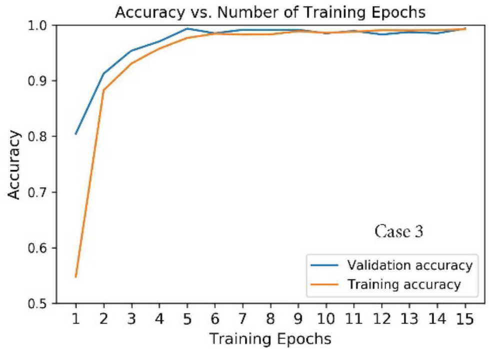
Accuracy vs. Number of Training Epochs
Accuracy
Training Epochs
Case 3
Validation accuracy
Training accuracy

(E)

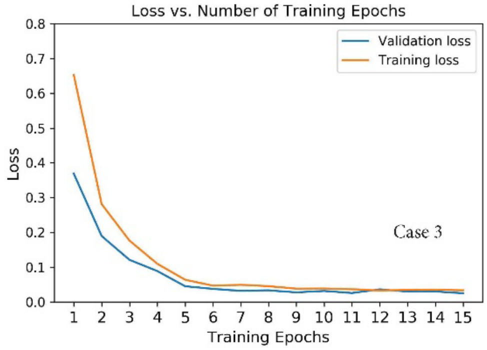
Loss vs. Number of Training Epochs
Loss
Training Epochs
Validation loss
Training loss
Case 3

(F)

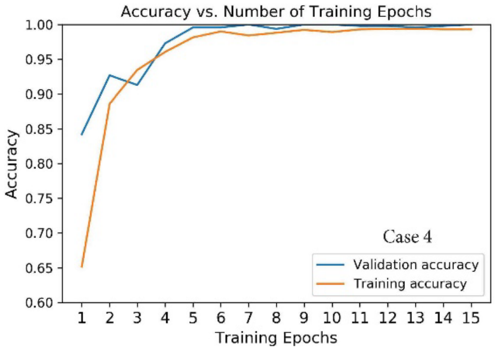
Accuracy vs. Number of Training Epochs
Accuracy
Training Epochs
Case 4
Validation accuracy
Training accuracy

(G)

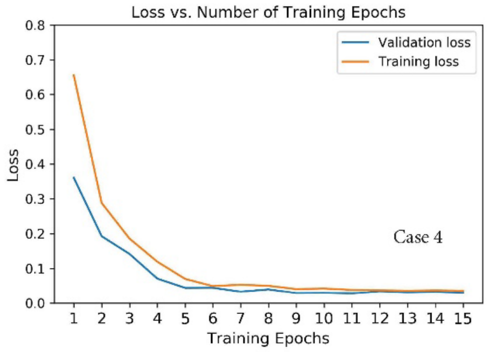
Loss vs. Number of Training Epochs
Loss
Training Epochs
Validation loss
Training loss
Case 4

(H)

Supplementary Figure 2. The learning curves of the CNN models Case 1 to Case 4 trained on Dataset 2 are shown. The cases of study presented different weights in the loss function, [weight in Category 1, weight in Category 2, weight in Category 3]. The plots (A) and (B) represent the learning curves of Case 1 [1,1,1], (C) and (D) Case 2 [1,10,1], (E) and (F) Case 3 [2,5,1] and (G) and (H) Case 4 [2,1,1].The left plots show training (orange solid line) and validation (blue solid line) accuracy versus the number of the epoch trained on Dataset 2. The right plots represent the loss versus the number of the epoch trained on Dataset 2.
